# Metoprolol-Associated Central Nervous System Complications

**DOI:** 10.7759/cureus.8236

**Published:** 2020-05-22

**Authors:** Rony Shah, Alina Babar, Amar Patel, Ronald Dortonne, Jeffrey Jordan

**Affiliations:** 1 Internal Medicine, HCA Citrus Memorial Hospital, Inverness, USA; 2 Internal Medicine, Citrus Memorial Hospital, Inverness, USA

**Keywords:** metoprolol toxicity, metoprolol side effects, cns side effects, metoprolol therapy, bizzare and vivid dreams, sleep disturbances, delirium, beta-blocker side effects

## Abstract

Metoprolol is a common medication used by the elderly because it is affordable and has proven to decrease mortality in cardiovascular disease. Multiple studies have reported central nervous system (CNS) side effects associated with use of beta-blockers. The risk of beta-blocker CNS side effects is directly associated with the lipophilic property of the drug.

We present the case of an 84-year-old male presented to the clinic complaining of increased confusion, fatigue, lightheadedness, nightmares, sleep disturbance, and gait problems for four weeks. The patient was evaluated for neurogenic and cardiogenic causes of his symptoms and both were ruled out. We believe that further review of his medical chart and medication reconciliation will lead us to the underlying cause of his symptoms.

Despite being an effective treatment option, there are risks associated with beta-blocker therapy. The most common symptoms are psychiatric conditions, bizarre and vivid dreams, sleep disturbances, delirium, psychosis, and visual hallucinations. Elderly patients who are started on beta-blockers require close monitoring for any adverse neurological symptoms.

## Introduction

Beta-blockers are widely used for the treatment of cardiovascular and noncardiovascular conditions. Metoprolol is a common medication used by the elderly because it is affordable and has proven to decrease mortality in cardiovascular disease such as congestive heart failure (CHF) and coronary artery disease (CAD). For years, beta-blockers have been known to cause central nervous system (CNS) side effects, such as psychiatric conditions, bizarre and vivid dreams, sleep disturbances, delirium, psychosis, and visual hallucinations [1]. The CNS side effects are associated with the lipophilic properties of beta-blockers, which allow them to rapidly penetrate the blood-brain barrier [1,2]. Highly lipophilic beta-blockers, such as propranolol, rapidly penetrate brain tissue compared to water-soluble beta-blockers, such as atenolol, which do not [3]. Metoprolol is a moderately lipophilic beta-blocker and if administered at clinical doses it has a low incidence of neuropsychiatric side effects [3,4]. Risk factors associated with these side effects are old age, impaired liver function, or pre-existing cognitive deficits [3]. We present a unique case of metoprolol-induced CNS complications.

## Case presentation

**Figure 1:**
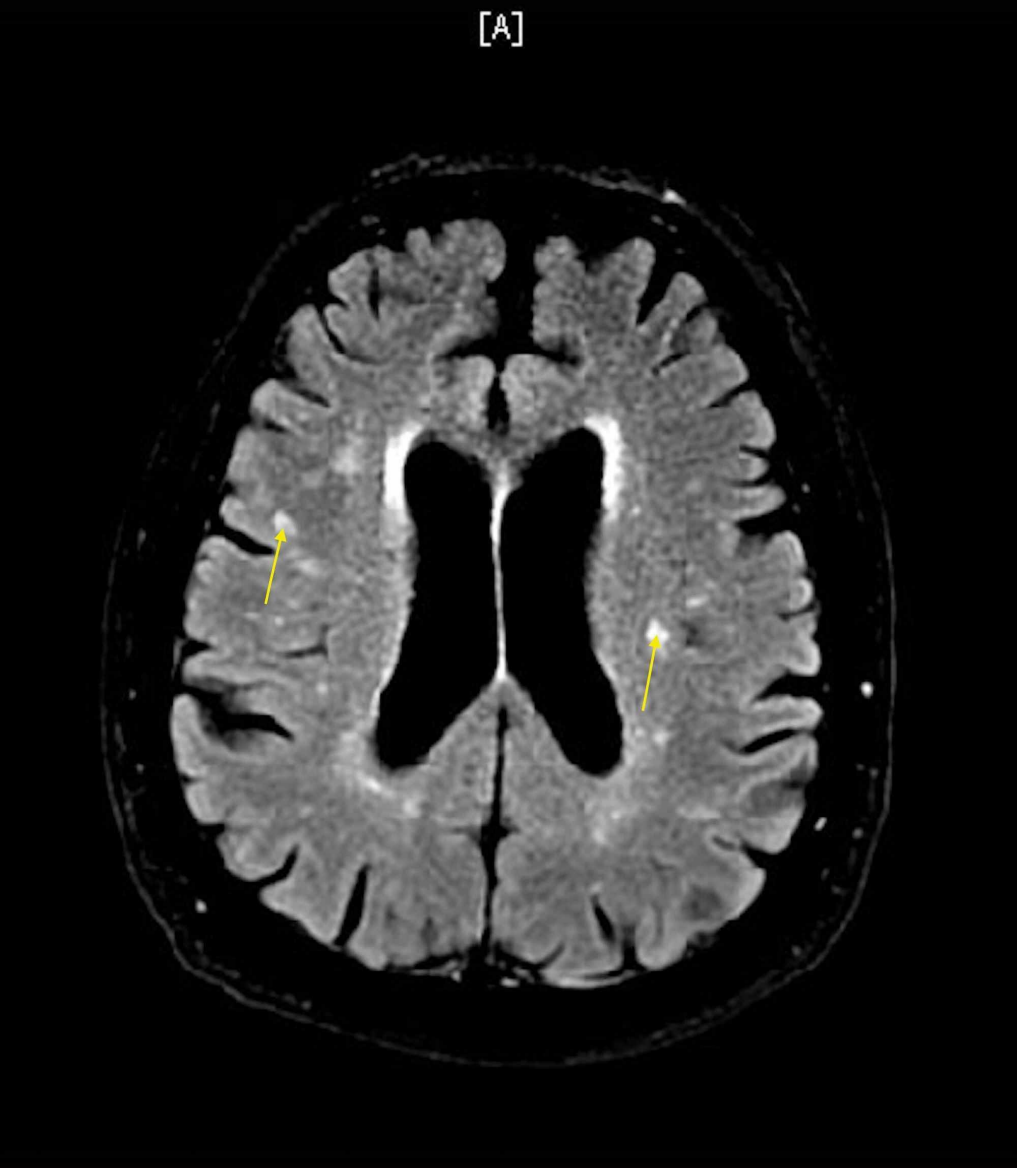
MRI brain without contrast showing white matter hyperintensities, consistent with chronic white matter ischemic changes

An 84-year-old male presented to the clinic complaining of increased confusion, fatigue, lightheadedness, nightmares, sleep disturbance, and balance problems for four weeks. He gave a past medical history of aortic valve replacement, hypertension, hyperlipidemia, benign prostate hyperplasia, Barrett’s esophagus, mild dementia, and chronic back pain. The patient was evaluated in an emergency room (ER) six weeks ago for lightheadedness and fatigue. Acute coronary syndrome and cerebrovascular accident were ruled out during the ER visit. Troponin (x2) was within normal range. Electrocardiogram (EKG) showed sinus rhythm with left anterior fasicicular block. Chest x-ray showed no cardiopulmonary abnormalities. MRI showed chronic ischemic white matter changes but no acute abnormalities (Figure 1). The patient’s symptoms were believed to be secondary to orthostatic hypotension; orthostatic vitals were supine blood pressure (BP) of 150/80 mmHg and standing BP of 130/70 mmHg. Adjustment to his BP medications was made, his amlodipine dose was decreased from 10 mg daily to 5 mg daily. The patient was evaluated by a cardiologist after being discharged from the ER. The cardiologist discontinued his amiodarone due to his ataxia, but continued his metoprolol. The patient was started on metoprolol and amiodarone for postoperative atrial fibrillation following aortic valve replacement nine months ago. He had mild improvement in his fatigue and lightheadedness following adjustment in his medications, but continued to have worsening confusion, nightmares, sleep disturbances, and ataxia which made him come to the clinic. He described his dreams as if someone was coming to attack him and he would wake up swinging because it felt so real. The patient mentioned that his symptoms begin soon after he takes his metoprolol every day. At that encounter, his metoprolol tartrate was decreased from 50 mg twice a day to 25 mg twice a day. The patient’s symptoms improved after decreasing his metoprolol dose. With improvement in symptoms, metoprolol was tapered off at his follow-up clinic visit. His metoprolol was decreased from 25 mg twice a day to 12.5 mg twice a day for seven days. The patient’s symptoms completely resolved, and his confusion improved during his follow-up visit.

## Discussion

Metoprolol is a safe and effective beta-adrenergic medication used to treat a variety of cardiovascular conditions. Since beta-blockers were introduced in clinical practice, they have been associated with CNS-related side effects [2]. The incidence of neuropsychiatric side effects in beta-blockers is associated with their lipophilicity, which is the ability of the drug to cross the blood-brain barrier [5]. Highly lipophilic agents, such as propranolol, have a higher incidence compared to more hydrophilic agents, such as atenolol. As mentioned previously, metoprolol is a moderately lipophilic agent with a brain/plasma concentration ratio 20 times higher than atenolol [5]. Highly lipid-soluble beta-blockers will be quickly and rapidly absorbed in the gastrointestinal tract and metabolized by the liver, and then widely distributed to all tissues [2]. In contrast, hydrophilic or water-soluble beta-blockers are not absorbed as well in the gastrointestinal tract or metabolized; they are excreted by the kidneys and less widely distributed to all the tissues [2]. The concentration of moderately and highly lipophilic beta-blockers in the cerebrospinal fluid (CSF) is approximately similar to the concentration in the plasma. However, for water-soluble beta-blockers, the concentration in the CSF is one-fifth to one-tenth of the plasma concentration [2]. A possible explanation of this is that water-soluble beta-blockers equilibrate slowly across the blood-brain barrier compared to moderate or highly lipophilic beta-blockers that equilibrate within minutes [2]. 

There is limited data available on the occurrence of CNS side effects with the administration of metoprolol [1]. Theoretically metoprolol can cause all of the CNS side effects associated with beta-blockers, such as psychiatric conditions, bizarre and vivid dreams, sleep disturbances, delirium, psychosis, and visual hallucinations [1]. Our patient was experiencing delirium, vivid and bizarre dreams, and sleep disturbances. A retrospective study by Gliebus and Lippa found that beta-blockers caused memory impairment in people with pre-existing cognitive impairment. It is believed the role of norepinephrine in memory is related to its role in signaling through hippocampal beta 1-adrenergic receptors. If the number of hippocampal neurons is reduced due to an underlying pathology such as Alzheimer’s dementia or ischemia and the adrenergic system is blocked by beta-blockers, it is believed that recently formed memories would be affected and the daily functioning of the patient is worse [6]. Our patient had mild dementia, which was most likely exacerbated by metoprolol use. Patients with cardiovascular disease have a greater risk of developing psychological disorders, such as depression and anxiety [2]. The use of beta-blockers in these patients is controversial due to its potential side effect of causing anxiety and depression despite well-established benefits [2]. The prevalence rates of depression and anxiety in patients with CHF were reported as 10%-60% and 11%-45% during literature review [7]. A study by Burkauskas et al. reported an association between beta-blocker use and psychological symptoms in patients with CAD [8]. The emotional stress from depression and anxiety can cause burnout syndrome which presents as fatigue and exhaustion [2]. Burnout syndrome is believed to be a combination of depression and anxiety disorders [9]. Our patient had a recent aortic valve replacement and developed postoperative atrial fibrillation. The recovery following cardiac surgery and development of a new-onset arrhythmia may have contributed to burnout syndrome in our patient. The use of metoprolol during this time period may have exacerbated his symptoms. 

A previous study by Goldner found an association between metoprolol and visual hallucinations [1]. Metoprolol-induced visual hallucinations may be underreported due to several factors, such as inability to connect the symptom to the drug or attribute the hallucinations to dreaming or nightmares [1]. Patients maybe too embarrassed to discuss the symptoms due to the fear it may be confused with mental illness or substance abuse, or physicians may fail to recognize the adverse drug effect and may consider it to be related to a pre-existing medical or psychiatric condition [1]. Sirois reported in his study that hallucinations due to metoprolol may be an isolated symptom or evolve into delirium in older patients with previous cognitive deficits [10]. Hallucinations caused by metoprolol typically stop within a few days after discontinuing the drug [1]. Previous studies have also reported bizarre dreams and hallucinations with hypnagogic features [3,11]. Our patient reported bizarre nightmares with feeling of someone coming to attack him and waking up sensing it was real. Cove-Smith and Kirk reported a significant increase in sleep disturbances and restless nights in patients on metoprolol compared to atenolol [3]. In our case, the patient reported restless nights and sleep disturbances as well. The withdrawal of lipophilic beta-blockers resulted in significant improvement in sleep, dreams, concentration, energy, memory, and anxiety [3]. Similar results were found in our case, but we recommend an observational study to find connections between metoprolol use and CNS side effects. 

## Conclusions

Beta-blockers are commonly prescribed to elderly patients for various medical conditions. They have proven to decrease the mortality of cardiovascular conditions, such as CAD and heart failure. As physicians we need to monitor our patients for any neurological changes after starting beta-blockers. On many occasions we may overlook neurological changes as part of the aging process in our elderly patients. Despite being an effective treatment option, there are risks associated with beta-blocker therapy.
